# Characteristics of Occlusal Force and Masticatory Performance in Female Patients Who Selected Implant Treatment for a Missing Mandibular Second Molar: A Retrospective Study

**DOI:** 10.3390/jfb16060211

**Published:** 2025-06-05

**Authors:** Takashi Abe, Motohiro Munakata, Takumi Yokoi, Kikue Yamaguchi, Daisuke Sato, Kazuyoshi Baba

**Affiliations:** 1 Department of Implant Dentistry, Showa University School of Dentistry, 2-1-1 Kita-senzoku, Ota-ku, Tokyo 145-8515, Japan; t.abe@dent.showa-u.ac.jp (T.A.); kyamaguchi@dent.showa-u.ac.jp (K.Y.); dsato.imp@dent.showa-u.ac.jp (D.S.); 2 Department of Prosthodontics Dentistry, Showa University School of Dentistry, 2-1-1 Kita-senzoku, Ota-ku, Tokyo 145-8515, Japan; t-yokoi@dent.showa-u.ac.jp (T.Y.); kazuyoshi@dent.showa-u.ac.jp (K.B.)

**Keywords:** dental implant, second molar, occlusal force, masticatory performance, single implant

## Abstract

Background: In this study, we aimed to investigate the association between a patient’s selection of implant treatment for a missing mandibular second molar and the magnitude of occlusal force, masticatory ability, mandibular morphology, and age before treatment intervention. Materials and Methods: We retrospectively assessed occlusal force, masticatory performance, and mandibular morphology in female patients who either selected or declined implant treatment for a missing unilateral mandibular second molar. Results: Thirty-three women (mean age of 56.1 ± 9.7 years) were divided into an implant treatment (IT) group and a no-treatment (NT) group. The IT group showed significantly higher occlusal force (*p* = 0.021 < 0.05), while masticatory performance and gonial angle demonstrated no significant difference. Conclusion: The IT group had significantly higher occlusal force, and age had no significant effect on it. Notably, masticatory performance in the IT group increased significantly with age (*p* = 0.047 < 0.05).

## 1. Introduction

Implant-prosthetic treatment for missing teeth has been established as a treatment capable of restoring high functionality and esthetics, and many studies have reported its high long-term predictability [1,2,3]. Additionally, alveolar bone resorption due to periodontitis and apical periodontitis often causes tooth extraction. Through the use of horizontal or vertical bone augmentation, maxillary sinus augmentation using guided bone regeneration, and short implants, the potential for implant treatment is expanding, even in the molar area. This region is susceptible to bone volume deficiency due to issues related to the anatomical morphology of the mandibular canal and maxillary sinus. Additionally, a systematic study revealed that the implant insertion process has been made simpler by alveolar ridge preservation, a technique that was recently developed to retain the alveolar crest in extraction sockets [4,5,6,7].

Particularly, implant-prosthetic treatment intervention for missing molar free-end cases is more effective than conventional removable partial dentures in terms of favorable restoration of the occlusal support area, the acquisition of occlusal force that is equivalent to that of natural teeth, a reduction in the burden on the remaining teeth, and lower caries risk. Moreover, numerous studies along with systematic reviews have demonstrated that it results in high patient satisfaction and improves oral-related quality of life (QOL) [8,9].

Conversely, many studies have reported complications caused by implant treatment, including biological complications, mainly due to bacterial infection, such as peri-implant inflammation and the failure of osseointegration, as well as mechanical complications, including the fracture of prosthetic devices due to excessive occlusal force, the loosening or fracture of screws, and the fracture of implant bodies [2,10,11,12,13]. Particularly, implant treatment for mandibular molars reportedly has a higher risk of causing biological and mechanical complications compared to those for other areas. The oral vestibule can become narrow due to a lack of keratinized mucosa, and plaque control is difficult as patients wear a prosthetic device that is difficult to clean, with excessive occlusal force applied at the same time [14,15]. Specifically, because it is a single crown, implant treatment for a single missing mandibular second molar is vulnerable to the fracture of the implant body and superstructure, screw loosening, and loss of osseointegration due to occlusal pressures and bruxism, among other factors.

Because of these risks, prosthetic treatment in the molar area, especially for the second molar, is avoided in some cases. Prosthetic treatment up to the first molar area has reportedly yielded satisfactory outcomes in terms of functionality and oral-related QOL [16,17,18,19]. Therefore, although implant treatment is now recognized as an option for prosthetic treatment for missing teeth, the second molar is the most distal area, and there is no established theory regarding whether prosthetic intervention should be provided to this region, which has a high risk of developing complications. Particularly for implant treatment for missing second molars, while it is difficult for dentists to judge whether they should provide the treatment intervention, they often encounter patients who desire implant treatment due to the chief complaint of masticatory difficulty.

In this study, we aimed to clarify the clinical characteristics of patients who request implant treatment for a missing second molar as a first step toward developing evidence-based guidelines for such cases. Specifically, we evaluated objective factors—maximal occlusal force, masticatory performance, mandibular morphology, and age—in female patients prior to treatment, and we compared these parameters between those who selected to receive implant placement and those who did not.

## 2. Materials and Methods

Measurements were obtained from patients with missing second molars. Patients were informed about the treatment options—partial denture, implant, and no prosthesis—as well as the advantages and disadvantages of each. Those who chose either an implant or no prosthesis were then compared. Additionally, patients were informed that they could withdraw from this study at any time without affecting their future treatment.

**Figure 1:**
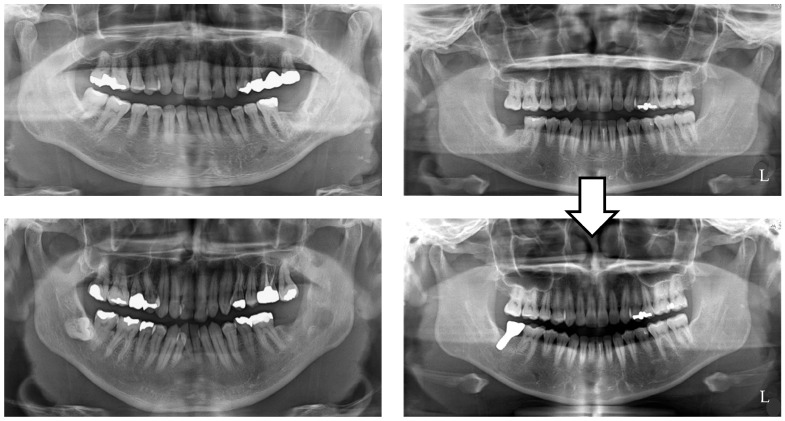
(**Left**) A representative patient in the NT group. (**Right**) A representative patient in the IT group (NT: no treatment; IT: implant treatment).

This study was retrospective and targeted female patients aged 40 years and older who had a missing unilateral mandibular second molar with an opposing maxillary second molar and visited the Showa University Dental Hospital Implant Center between April 2021 and April 2023. The study group (IT group) consisted of patients who experienced masticatory difficulty and had selected implant treatment. The control group (NT group) consisted of patients who did not select prosthetic intervention for a missing mandibular second molar (Figure 1). All patients in both groups were selected after their doctors had fully explained the advantages and disadvantages of implant treatment interventions for second molars.

The exclusion criteria were as follows:

1. Patients who had untreated, missing teeth in areas other than the one planned for implant treatment.
2. Patients who were undergoing implant treatment in other areas.
3. Patients with at least one erupted third molar on the left or right side of the upper or lower jaw.
4. Patients who had lost a mandibular second molar within the past 3 months.
5. Patients with insufficient bone volume, in whom the distance to the mandibular canal of the second molar where an implant was placed was ≤10 mm.
6. Patients wearing a removable prosthetic device.
7. Patients with temporomandibular disorders.
8. Patients with mobile teeth due to periodontitis.
9. Patients who had difficulty communicating due to mental illness.
10. Patients with bone metabolic disease (such as osteoporosis and rheumatoid arthritis).
11. Patients who had previously received radiation therapy for the jawbone.
12. Patients taking bone resorption inhibitors.

The items examined were occlusal force, masticatory performance, and gonial angle. These were measured as described below, with all measurements in the IT group taken before implant placement surgery. Furthermore, the NT group underwent maintenance every 3 to 6 months to check the extension of the opposing teeth, changes in occlusion, and plaque control to prevent periodontal disease.

**Figure 2:**
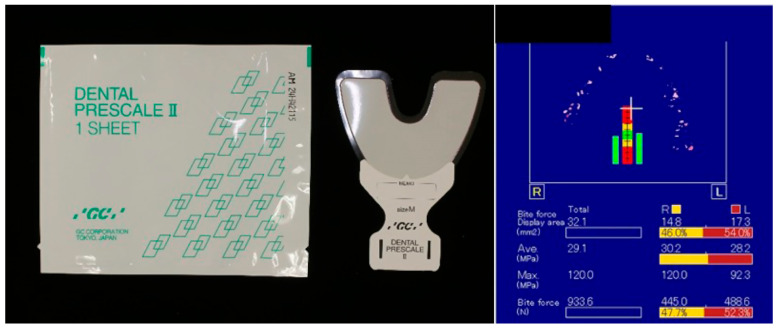
Measurement of maximal occlusal force.

(1) Maximal occlusal force (Figure 2)

A film designed for an occlusal force measuring system (Dental Prescale II^®^; GC Corp., Tokyo, Japan) was placed over the entire dentition, and the participants were instructed to clench their teeth with maximum force in the intercuspal position for 3 s. Two measurements were taken, with an interval of 1 min. The maximal occlusal force was analyzed using a reading scanner (GT-X830; Seiko Epson Corp., Nagano, Japan) and analysis software (Bite Force Analyzer; GC Corp., Tokyo, Japan). These measurements were conducted by the same examiner, and the maximal occlusal force was taken as the mean value of the two.

**Figure 3:**
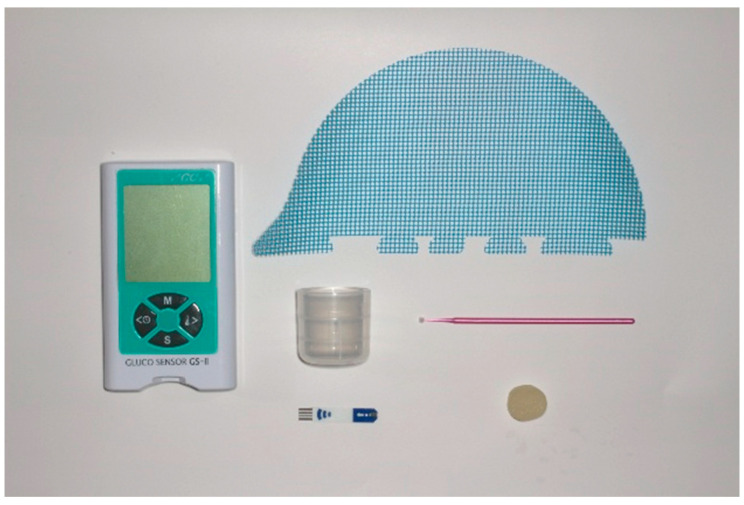
The glucose-measuring device used to determine masticatory performance.

(2) Masticatory performance (Figure 3)

The participants were instructed to place a glucose-containing gummy jelly (Glucolumn; GC Corp., Tokyo, Japan) in their mouths and masticate for 20 s without swallowing saliva or the jelly. After 20 s, the participants held 10 mL of water in their mouths and spat out the masticated jelly and water together into a cup equipped with a filter mesh. The amount of glucose eluted from the filtrate in the cup was measured using a glucose-measuring device (Gluco Sensor GS-II; GC Corp., Tokyo, Japan), and masticatory performance was calculated [20,21,22].

**Figure 4:**
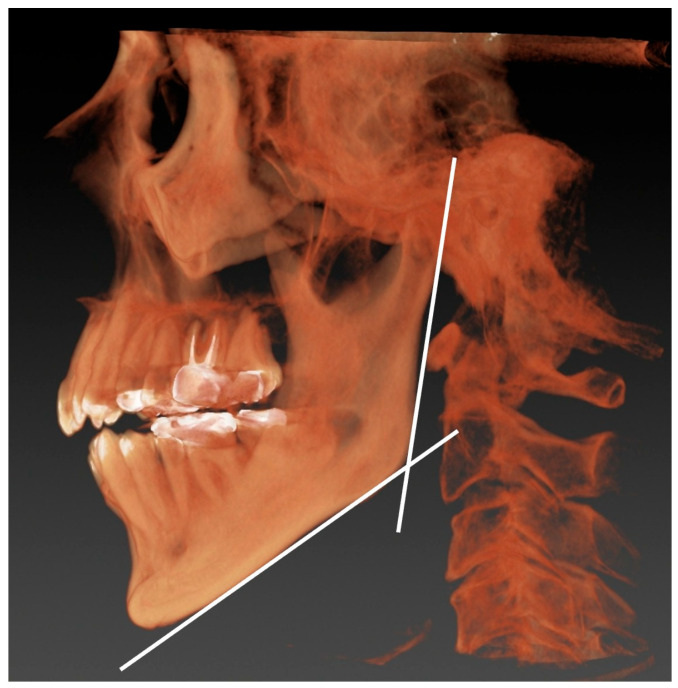
Measurement of the gonial angle. The angle formed at the intersection of the ramus and mandibular line on a 3D image was measured.

(3) Gonial angle (Figure 4)

The gonial angle was considered an indicator of patients’ occlusal forcefulness, and it was measured according to the method described by Anderson et al. [23]. The images were acquired using a dental CBCT (KaVo 3D Exam; KaVo Dental Systems, Biberach, Germany). The imaging parameters were set to 120 kVp, 5 mA, an acquisition time of 8.9 s, an axial slice thickness of 0.25 mm, and an isotropic voxel size of 16 × 16 cm. Prior to any measurement, the cone-beam computed tomography (CBCT) volume was adjusted such that the Frankfort plane (eye–ear plane) and interorbital line were parallel to the horizontal plane, and any right–left rotations were corrected. The gonial angle, as conventionally described, is that between the intersection of the ramus and the mandibular lines. All the images were recorded in Digital Imaging and Communications in Medicine (DICOM) format, and the DICOM data were analyzed using simulation software (Invivo5: Anatomage, Santa Clara, CA, USA).

The gonial angle was taken as the mean value of the left and right sides.

One radiologist with over 10 years of experience conducted the gonial angle measurements based on the CBCT.

### Statistical Analysis

Each item was compared between the IT and NT groups, and the effect of age was examined. A statistical analysis was conducted using the Mann–Whitney U-test and Spearman’s rank correlation coefficient (*p* < 0.05).

The Ethics Committee of Showa University approved this study protocol (approval number DH2018-009; approval date: 14 August 2018).

## 3. Results

The participants included 33 females (mean age: 55.1 ± 7.1 years), with 21 in the IT group (mean age: 54.5 ± 7.1 years) and 12 in the NT group (mean age: 56.0 ± 7.8 years). The measurement results of the items examined are shown in Table 1.

1. Maximal occlusal force

The mean occlusal force was 739.2 ± 374.9 N in the IT group and 584.1 ± 293.4 N in the NT group, with the IT group showing significantly higher occlusal force (*p* = 0.021 < 0.05). The effect of age was small in both the IT and NT groups, with no significant difference between the groups (IT group: *r* = −0.16; *p* = 0.315; NT group: *r* = 0.65; *p* = 0.117) (Table 2).

2. Masticatory performance

The mean masticatory performance was 235.8 ± 127.1 mg/dL in the IT group and 215.5 ± 76.5 mg/dL in the NT group, with the IT group showing higher values. However, there was no significant difference between the groups (*p* = 0.51). Additionally, masticatory performance in the IT group showed a positive correlative relationship with age (*r* = 0.53; *p* = 0.047 < 0.05) (Table 2).

3. Gonial angle

The mean gonial angle was 125.0 ± 5.1° in the IT group and 121.9 ± 9.8° in the NT group, with no significant difference between them (*p* = 0.314). There was no statistically significant difference in correlation with age for either the IT group or the NT group (Table 2).

## 4. Discussion

The concept of providing prosthetic intervention for missing molars has been examined in several studies. Regarding removable prosthetic intervention, Walter et al. investigated the periodontal health of patients who had lost their molars by conducting a 10-year comparative analysis between patients wearing partial removable denture prostheses and those receiving no intervention. The latter showed favorable outcomes in all parameters relating to remaining teeth (clinical attachment loss, bleeding on probing, and plaque index) [24]. Furthermore, as regards providing implant-prosthetic intervention for a missing second molar, it has been reported in some studies that implant treatment is more effective than other prosthetic treatments. Contrastingly, others have opined that implant-prosthetic intervention in this region is unnecessary because of its higher risk of developing complications compared to that in other areas, and this thereby remains a controversial topic [25,26].

In clinical practice, treatment using the all-on-four concept, which does not involve prosthetic treatment for the second molar area, can also achieve favorable long-term outcomes [27,28,29]. Malo et al. reported high cumulative survival and success rates of 93% and 91.7%, respectively, after a long-term follow-up of 10 years and up to 18 years. Based on the all-on-four concept, which is characterized by prosthetic appliances up to the first molar without a second molar, they examined the prognosis of fixed full-arch prosthetic treatment for the mandible. Additionally, they concluded that the treatment method was very predictive, with low marginal bone loss values after 10 and 15 years following treatment of 1.72 mm and 2.32 mm, respectively [27]. According to Uesugi et al.’s longitudinal study of Japanese patients with 3–17-year survival rates, the cumulative survival rate for the maxilla was 94.4% at the patient level and 97.4% at the implant level, while that for the mandible was 96.7% at the patient level and 98.9% at the implant level [28]. The results indicated that the treatment of the mandible was a successful therapeutic strategy for Japanese patients. Additionally, there was increased enjoyment and quality of life associated with oral health in the edentulous individuals who received full dentures supported by all-on-four implants.

Patients whose rehabilitation was based on the all-on-four concept had excellent oral health-related quality of life and satisfaction, according to a systematic review of the concept that used the oral health impact profile (OHIP) and visual analog scale (VAS) assessment [29]. Therefore, it has been demonstrated that even with prosthetic appliances up to the first molar without a second molar, good long-term prognosis may be achieved, and patient satisfaction and quality of life are high when it comes to full-arch prosthetic treatment with implants.

Regarding treatment planning for older adults with periodontal disease, Curtis et al. noted that the use of removable partial dentures can pose problems, including increased plaque accumulation and caries risk in proximal plates and rests. Moreover, they emphasized the importance of keratinized mucosa and crown contours of prosthetic devices in risk assessment when selecting implant treatment. They further cited difficulty in plaque removal due to keratinized tissue with a size of <2 mm and difficulty in oral hygiene access as factors [30]. Furthermore, patients undergoing implant treatment require professional maintenance, and the incidence rate of peri-implantitis has been reported to differ significantly based on the presence or absence of hospital visits for maintenance purposes [31,32,33].

In a study by Horibe et al., which used a device similar to that employed in this study, the occlusal force in 391 males and females aged 65 years and older was measured to be 546.5 ± 418.6 N [34]. Additionally, Shiga et al. investigated the effect of aging on the occlusal force of patients with natural dentition by comparing three age groups—a young group (20–39 years), a middle group (40–59 years), and an old group (60 years and over)—and they reported a significant decrease in occlusal force among those aged 60 years and older in both gender [35]. Moreover, a similar study conducted by Sano et al. reported that both sexes showed significant decreases in occlusal force with aging, with a stronger tendency in females, and that the young group aged 21–49 years had a maximal occlusal force of 495.5 ± 144.3 N [20]. In this study, the IT group demonstrated a significantly high occlusal force of 739.2 ± 374.9 N, with no effect of age, which was different from the results of the studies by Horibe et al., Shiga et al., and Sano et al. These results suggest that the patients who selected implant treatment for their second molars had high occlusal forces at any age.

Regarding masticatory function, Shiga et al. examined the relationship of masticatory performance with gender difference and age using a similar device to that in this study. Masticatory performance was significantly higher in males than in females, and there was no effect of aging in either gender [21]. Additionally, Sano et al. examined the association among gender difference, occlusal force, and masticatory performance, reporting that occlusal force and masticatory performance were significantly lower in females (208.8 mg/dL in the young group and 197.4 mg/dL in the older group), and they were positively correlated with each other in both genders [20]. Furthermore, Nimura et al. examined the association among occlusal force, masticatory function, and physical constitution (height, weight, and body mass index (BMI)), showing that occlusal force and masticatory performance were significantly lower in females (452.9 N and 194.4 mg/dL, respectively) and that although occlusal force was associated with physical constitution (especially weight), there was no association with masticatory performance [36]. Additionally, Yokoi et al. conducted a functional assessment of patients with a missing second molar in either the upper or lower jaw using a similar device to that in this study, and they analyzed the differences between the implant treatment intervention group and the untreated group. They concluded that there was no difference in masticatory performance or occlusal force between the two groups [37]. In this study, the IT group showed a masticatory performance of 235.8 ± 127.1 mg/dL, which was significantly higher than those reported in other studies, and it showed a significant improvement with age. The results suggest that the patients who selected implant treatment for their second molars also exhibited high masticatory ability, which was not undermined by age. This could be attributed to the small sample of patients that had small numbers of missing teeth, good oral hygiene, and affluent socio-economic status.

Regarding the gonial angle, Miwa et al. examined the relationship between this and the maximum occlusal force in young men and women (average age of 24.6 years) using panoramic radiographs. They reported that the maximum occlusal force in men was significantly higher than that in women, and though there was no gender difference in the gonial angle in a multiple regression analysis, a strong negative correlation with the maximum occlusal force was observed [38]. Similarly, Bakan et al. measured the gonial angle using CBCT and examined differences between age and gender, and they reported that there were no differences regarding each of these three factors [39]. In this study, although the IT group had a significantly higher maximum occlusal force, there was no difference in the gonial angle, and age had no effect, consistently with the findings of Bakan et al. The discrepancy with the results from Miwa et al. was possibly due to the difference in measurement methods, as this study targeted relatively older people in their 40s to 60s, with a narrow age range within the patient groups. Recently, Akter et al. investigated the relationship between gonial angle, which is considered the indicator of the magnitude of occlusal force, and late implant loss in order to determine whether occlusal overload is a risk factor for late implant loss. The results of the multivariate analysis reported a significant correlation between a decrease in the gonial angle and late implant loss [40]. Therefore, practitioners should be cautious when implant therapy is planned for a missing mandibular second molar, which is subject to a single crown and high occlusal forces, for patients with smaller gonial angles.

Greenstein et al. created guidelines for the management of missing second molars, stating the following: (1) The decision to replace a missing second molar with implant treatment is optional and should be judged based on patient-specific criteria. (2) Because the over-eruption of an unopposed second molar tends to occur early after an extraction, it is prudent to perform implant treatment as early as possible if it is planned. (3) The bone volume should be thoroughly examined using CT images if implant treatment is considered. (4) Routinely replacing a second molar due to reduced masticatory efficiency is unnecessary. Generally, patients function well with first-molar occlusion. Continued monitoring is recommended for asymptomatic patients who are not manifesting any occlusal alterations. (5) Position changes in opposing teeth can be avoided through preventive and conservative measures (e.g., the bonding of the second molar to the first molar and fabrication of a retainer). (6) Because opposing teeth with periodontal disease tend to over-erupt more than healthy molars, increased monitoring is needed to prevent this [41]. In addition, Esposito et al. compared the prognoses of short implants (4.0 mm long) and normal implants (8.5 mm long or longer) in an RCT investigating the efficacy of short implants for posterior jaws and reported comparable survival rates, albeit for a shorter time period. The results showed that the survival rates were comparable between short implants and normal implants [42]. In this respect, an individual with a missing second molar can be a good candidate for implant treatment even with limited bone height.

The findings in this study suggest that patients who report masticatory discomfort or difficulty due to a missing mandibular second molar and who actively seek treatment are often characterized by possessing a relatively large occlusal force and superior masticatory performance compared to those who do not pursue such treatment. This observation implies that individuals with higher functional demands—those who naturally generate stronger occlusal loads and exhibit more efficient chewing—may be more sensitive to the absence of posterior occlusal support, even in areas that are sometimes considered less critical, such as the second molar region.

It is therefore speculated that these patients perceive the functional deficit caused by the missing second molar more acutely, potentially due to their habitual reliance on a full complement of posterior teeth to maintain their high chewing efficiency. As a result, they may experience a more pronounced decline in oral function when a second molar is lost, leading to a stronger motivation to seek implant-based restoration. This inclination suggests that, despite general trends showing lower prosthetic demand for second molars, there exists a subset of patients with high occlusal capabilities who regard the second molar as essential for maintaining optimal function and comfort in daily mastication.

Based on the lack of an effect of age, we believe that sufficient informed consent is needed from patients indicated for implant treatment, covering issues such as the presence of keratinized mucosa, the ability to control plaque, the selection of superstructure materials, periodic occlusal adjustment, the fabrication of a mouthpiece as necessary, and bone volume. Additionally, for patients who have not received implant treatment for missing mandibular second molars, regular maintenance is essential for monitoring the extension of opposing teeth, changes in occlusion, and plaque control to prevent periodontal disease, as shown in the guidelines of Greenstein et al. [41].

Based on previous reports of gender differences in occlusal force and masticatory performance, only females were targeted in this study [20,21,43]. It is reported that females generally show lower occlusal force and masticatory performance compared to males, with a tendency towards a larger gonial angle. We also used bone volume factors as exclusion criteria to ensure that the evaluations were based on the same criteria. The final sample size of this study was smaller than originally anticipated, primarily due to difficulties encountered during patient recruitment. Despite thorough preparation and outreach efforts, several factors contributed to the lower-than-expected enrolment. These included a limited pool of eligible participants based on the inclusion and exclusion criteria, a relatively short recruitment period, and, in some cases, patient hesitancy to participate in clinical research. Consequently, the reduced sample size may limit the statistical power of this study, and the results should be interpreted with caution, particularly in terms of their generalizability and robustness. Future studies with a larger sample size will be necessary to confirm and expand upon the findings presented here. In addition, patients’ socio-economic status was not included in the analysis as one of the determinants of treatment selection because we could not retrospectively identify any variety in this among the participants. Nevertheless, this can be a potential determinant of treatment selection, and so it should be scrutinized and included as a parameter in any future prospective studies. Previous studies have suggested that occlusal force and masticatory performance are associated with physical constitution (including height, weight, and BMI), the period after tooth extraction, and the presence or absence of bruxism, angle classification, and masseter muscle thickness [44], which we believe require further investigation. As this study included patients with systemic diseases, such as controlled diabetes and cardiovascular disease, other than mental illness and bone metabolic disease, the impacts of these and their associated medications were not examined.

In this study, we focused exclusively on females, limiting generalizability across gender. In addition, bruxism, BMI, and muscle thickness, which are factors known to influence bite force, were not recorded and may have impacted the outcomes.

Additionally, since this study involved Japanese participants, it is possible that their dietary habits, culture, and preferences could have affected their decision to choose implant treatment for a single missing second molar, yielding different findings from research completed in the U.S. and Europe. The necessity of implant treatment for a missing mandibular second molar requires a sufficiently detailed study, including a survey using food questionnaires to assess the pre- and post-operative differences caused by implant treatment, because the same effects on patient satisfaction and quality of life can be taken into consideration.

In our future research, we would like to increase the number of patients and include males to investigate the effects of gender and age. Additionally, we will aim to investigate long-term prognoses with and without treatment intervention and examine the adequacy of implant treatment for a missing mandibular second molar.

## 5. Conclusions

Based on the results of this study, the following conclusions were drawn about the characteristics of the patients who selected implant treatment for a missing mandibular second molar: The IT group had a significantly higher occlusal force, with no effect of age. There was no statistically significant difference in masticatory performance between the IT and NT groups; however, the IT group showed a significant improvement with age. The effect of the gonial angle was small.

## Figures and Tables

**Table 1:** Measurement results.

	IT Group	NT Group	*p*-Value
Age	54.5 ± 7.1 years	56.0 ± 7.8 years	0.78
Maximal occlusal force	739.2 ± 374.9 N	584.1 ± 293.4 N	0.021 < 0.05
Masticatory performance	235.8 ± 127.1 mg/dL	215.5 ± 76.5 mg/dL	0.51
Gonial angle	125.0 ± 5.1°	121.9 ± 9.8°	0.314

NT: no-treatment; IT: implant treatment.

**Table 2:** Relationship with age.

	*r*	*p*
Maximal occlusal force		
IT group	−0.16	0.316
NT group	0.65	0.117
Masticatory performance		
IT group	0.53	0.047 < 0.05
NT group	0.13	0.39
Gonial angle		
IT group	0.16	0.248
NT group	0.65	0.159

NT: no-treatment; IT: implant treatment.

## Data Availability

The raw data supporting the conclusions of this article will be made available by the corresponding author on request.
